# Using time-dependent reproduction number to predict turning points of COVID-19 outbreak in Dalian, Liaoning province, China

**DOI:** 10.1186/s12879-022-07911-4

**Published:** 2022-12-10

**Authors:** Qingyu An, Jun Wu, Jin jian Bai, Xiaofeng Li

**Affiliations:** 1 Dalian Center for Disease Control and Prevention, Liaoning Province Dalian, People’s Republic of China 116023; 2 School of Public Health, Dalian Medical University, Dalian, 116044 Liaoning Province China

**Keywords:** COVID-19, ${R_{t}}$, Predict, Bayesion framework, Exponential trend equation

## Abstract

**Objectives:**

To forecast the development trend of current outbreak in Dalian, mainly to predict turning points of COVID-19 outbreak in Dalian, Liaoning province, China, the results can be used to provide a scientific reference for timely adjustment of prevention and control strategies.

**Methods:**

During the outbreak, Bayesian framework was used to calculated the time-dependent reproduction number (${R_{t}}$), and then above acquired ${R_{t}}$ and exponential trend equation were used to establish the prediction model, through the model, predict the ${R_{t}}$ value of following data and know when ${R_{t}}$ smaller than 1.

**Results:**

From July 22 to August 5, 2020, and from March 14 to April 2, 2022, 92 and 632 confirmed cases and asymptomatic infected cases of COVID-19 were reported (324 males and 400 females) in Dalian. The R square for exponential trend equation were 0.982 and 0.980, respectively which fit the ${R_{t}}$ with illness onset between July 19 to July 28, 2020 and between March 5 to March 17, 2022. According to the result of prediction, under the current strength of prevention and control, the ${R_{t}}$ of COVID-19 will drop below 1 till August 2, 2020 and March 26, 2022, respectively in Dalian, one day earlier or later than the actual date. That is, the turning point of the COVID-19 outbreak in Dalian, Liaoning province, China will occur on August 2, 2020 and March 26, 2022.

**Conclusions:**

Using time-dependent reproduction number values to predict turning points of COVID-19 outbreak in Dalian, Liaoning province, China was effective and reliable on the whole, and the results can be used to establish a sensitive early warning mechanism to guide the timely adjustment of COVID-19 prevention and control strategies.

**Supplementary Information:**

The online version contains supplementary material available at 10.1186/s12879-022-07911-4.

## Introduction

On December 31, 2019, an outbreak of pneumonia of unknown cause occurred in Wuhan, Hubei Province, China. Subsequently, on January 10, 2020, WHO confirmed that it was caused by a new novel Coronavirus [1]. On January 20, 2020, the national health commission of the people’s republic of China had listernational concern, and declared this public health emergency a pandemic on March 11, 2020[2, 3].

In Dalian, Liaoning province, China reported its first imported COVID-19 case on January 20, 2020. As of 24:00 Beijing time on July 21, 2020, 30 confirmed COVID-19 cases had been reported, including 12 imported cases from overseas [4]. During the period of above time, although there had been local secondary cases and epidemic cluster cases, no outbreak had occurred. On 22 July, 2020, and 14 March two COVID-19 outbreaks occurred in Dalian [5, 6].

In this study, we attempted to through calculate the time-dependent reproduction number (${R_{t}}$) to assess whether current control efforts were effective or whether additional interventions were required,meanwhile, established prediction model by used ${R_{t}}$ as basic data, and trying to predict the turning point of COVID-19 outbreak in Dalian, Liaoning province, China, the results can be used to provide a theoretical basis for epidemic prevention and control relevant policy formulation.

## Materials and methods

### Study area

Dalian is the main coastal city of Liaoning Province, China and a major tourist city located at 38°43′–40°10′N latitude and 120°58′–123°31′E longitude [7]. By 2018, there were 5.952 million registered urban residents [8].

### Data collection

In China, COVID-19 is a notifiable infectious disease, which is needed when doctors diagnose a case in the course of diagnosis and treatment,the clinicians were required to report COVID-19 cases through the China information system for disease control and prevention within 2 h. The COVID-19 cases included suspected case, confirmed case and asymptomatic infected case [9]. The illness onset time of the case were derived from epidemiological surveys.

### Data analysis

#### Time-dependent reproduction number (${R_{t}}$)

Time-dependent reproduction number ${R_{t}}$ refers to the number of new cases that can be caused by an average case at time ${t}$. ${t}$ is a unit of time, which can be hours, days, weeks, and so on.

Suppose that the infectiousness over time after novel Coronavirus infection is unrelated to the specific date. The time-dependent reproduction number (${R_{t}}$) is defined as the ratio of the number of new locally infected cases at time ${t}$(${I_{t}}$), and the total infection potential across all infected individuals at time ${t}$(${\Lambda_{t}}$). If there is a single serial interval distribution ${w_{s}}$ (s = 1, 2, …), representing the probability of a secondary case arising a time period ${s}$ after the primary case, each incident case that appeared at a previous time step ${t - s}$ contributes to the current infectiousness at a relative level given by $w_{s}$.

Therefore conditional on ${w_{s}}$, ${\Lambda_{t}}$ can be computed as:

1 $${\Lambda_{t}} (w_{s}) = \sum\limits_{s = 1}^{t} {{I_{t - s}} {w_{s}}} ,$$

$\Sigma$ denote the sum operation. The Poisson equation is used to fit the propagation process. The probability of observing ${I_{t}}$ cases at time step t is:

2 $$P(I_{t} |I_{0}, {I_{1}}, \ldots ,{I_{t - 1}} ,{w_{s}} ,{R_{t}} ) = \frac{{({R_{t}} {\Lambda_{t}} ({w_{s}})){I_{t}} \exp (- {R_{t}} {\Lambda_{t}} ({w_{s}}))}}{{I_{t}} !},$$

where exp denote an exponential function based on the natural constant e. We make the assumption that the reproduction number is constant over a time period [t − τ, t], with τ representing the length of the time window over which ${R_{t}}$ is estimated. The probability of observing the local incidence ${I_{t - \tau}}$, ${I_{t - \tau + 1}} , \ldots ,{I_{t}}$ during this time period, given the reproduction number ${R_{t}}$ and conditional on the previous incidence data ${I_{0}}, {I_{1}} , \ldots ,{I_{t - \tau - 1}}$, is

3 $$P({I_{t - \tau + 1}} , \ldots ,{I_{t}} \left| {{I_{0}} , \ldots ,} \right. {I_{t - \tau - 1}} ,{I_{t - \tau }} ,{w_{s}} ,{R_{t}} ) = \prod\limits_{k = t - \tau }^{t} {\frac{{(R_{t} {\Lambda_{k}} (w_{s} )){I_{k}} \exp ( - {R_{t}} {\Lambda_{k}} (w_{s} ))}}{{I_{k}} !}} ,$$

$\Pi$ denote the quadrature operation. Using a Bayesian framework with a gamma distributed prior for ${R_{t}}$, the posterior distribution of ${R_{t}}$ given past incidence data and conditional on the serial interval distribution, ${w_{s}}$, is

4 $$\begin{aligned} & P({R_t}\left| {{I_0},{I_1},{I_2}, \ldots ,{I_{t - \tau - 1}},{I_{t - \tau }},} \right.{I_t}_{ - \tau + 1}, \ldots ,{I_t},{w_s})\\ & \quad \propto P({I_t}_{ - \tau + 1}, \ldots ,{I_t}\left| {I_{0}, \ldots ,{I_{t - \tau - 1}},} \right.{I_{t - \tau }},{w_s},{R_t})P({R_t})\\ & \quad= \left( {\prod\limits_{k = t - \tau }^t {\frac{{({R_t}{\Lambda _k}({W_s})){I_k}\exp ( - {R_t} {\Lambda_k}({w_s}))}}{{{I_k}!}}} } \right)\left( {\frac{{{R_t}^{a - 1}\exp ( - {\frac{{{R_t}}}{b})}}}{{\Gamma (a){b^a}}}} \right)\\ & \quad\propto {R_t}^a + \sum\nolimits_{k = t - \tau}^t {{I_k} - 1\exp \left( { - {R_t}\left( {\sum\limits_{k = t - \tau }^t {{\Lambda _k}({w_s}) + {\frac{1}{b}}} } \right)} \right)} \times \prod\limits_{k = t - \tau }^t {\frac{{{\Lambda _k}({w_s}){I_k}}}{{I_k}!}},\end{aligned}$$

$\Gamma$ denote the gamma function, a and b is the shape and scale parameters of the gamma distributed prior for $R_{t}$. We use a gamma distributed prior, conjugate to the Poisson likelihood, to obtain an analytical formulation of the posterior distribution of $R_{t}$. According to the expression above, the posterior distribution for $R_{t}$ given the incidence data, conditional on the serial interval distribution $Ws$, is a gamma distribution with shape parameter $a + \sum\nolimits_{k = t - \tau }^{t} {I_{k} }$ and scale parameter $\frac{1}{{\sum\nolimits_{k = t - \tau }^{\tau } {\Lambda_{k} (w_{s} ) + 1/b} }}$ [14].

In this study, we assumed that the serial interval was (7.5 ± 3.4) days based on the epidemiological data reported by Li et al. [10], and the length of the time window was 7 days. R version 4.0.2 was used for data analysis. A p-value < 0.05 was considered statistically significant.

### Exponential trend equation

When COVID-19 outbreak occurred, the biggest concern for policy makers was when the outbreak would reach the peak, and when the outbreak would end, based on the current prevention and control efforts. In this study we focused to solve the latter problem.

The calculation of $R_{t}$ uses the onset time of case, and the earliest onset time of cases in two outbreaks are July 9 and March 5. In this study, a section of data with a declining incidence trend was selected as the basic data to fit the exponential trend equation, and the following value of $R_{t}$ with illness onset data were used to evaluate the quality of forecasting.

In this study, the illness onset time of the case can be divided into two types: to confirmed case, the illness onset date obtain through epidemiology interviews, and to asymptomatic infected case, the illness onset date equal to the date of diagnosis.

The exponential trend equation can be expressed as:

5 $$Y = {ab^{t}} ,$$

where $a$ represent the trend value at $t = 0$, $b$ represent the average growth rate,$t$ denote the number of time series. The statistical significance of the relationship depended on the value of regression coefficient R square [11]. In general, R square value greater than 0.85 can be considered as a good fitting effect and can be used for prediction.

The criterion for comparing the predictive ability of the model was the average relative error, defined as:

6 $$e = \frac{1}{n}\sum\limits_{t = 1}^{n} {\left[ {\frac{{(xt - \hat{x}t)}}{xt} \times 100\% } \right]} ,$$

where $xt$ and $\hat{x}t$ denote observed and fitted values for that point in time, $n$ denote the total number of time series, $e$ denote the value of average relative error. Microsoft Excel statistical package was used for data analysis.

All methods were carried out in accordance with relevant guidelines.

## Results

### Descriptive analysis

The two COVID-19 outbreaks occurred in Dalian lasted from July 22 to August 5, 2020, and from March 14 to April 2, 2022. During these period, 92 and 632 confirmed cases and asymptomatic infected cases of COVID-19 (324 males and 400 females), respectively were reported in Dalian, Liaoning Province, China. The cases were distributed in six of the 12 districts (Table 1). The median age of the patients was 40 years (range: 26 days to 89 years).

**Table 1:** The numbers of reported cases during two COVID-19 outbreaks in Dalian

District	Cumulative number of cases	Cumulative incidence rate (/100,000)
Jinzhou	589	38.111
Ganjingzi	77	5.017
Pulandian	49	7.782
Wafangdian	6	0.733
Xigang	2	0.655
Zhongshan	1	0.257
Overall	724	9.717

### Estimated the value of time-dependent reproduction number ($R_{t}$)

**Fig. 1:**
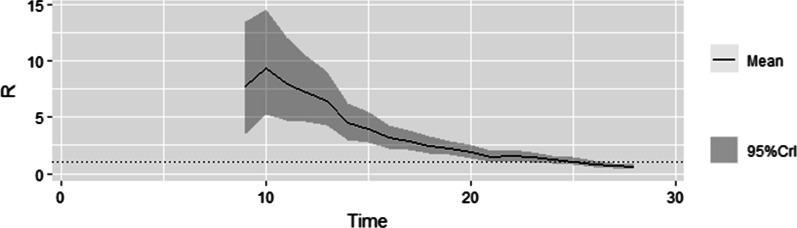
The distribution of likely values of time-dependent reproductive number during COVID-19 outbreak in Dalian during July 22 to August 5, 2020

The COVID-19 outbreak during July 22 to August 5, 2020: The calculation of $R_{t}$ uses the onset time of case, and the earliest onset time of cases in this outbreak is July 9. The mean reproduction number estimate for the first seven days of the outbreak namely during July 9 to July 15 was 7.687 (95% CI:3.515–13.463). The maximum value of R_t_ was 9.308 (95% CI:5.210–14.576), which was observed on July 18. Subsequently, as the strengthening of the prevention and control strategies, the R_t_ trend began to decline (Fig. 1).

**Fig. 2:**
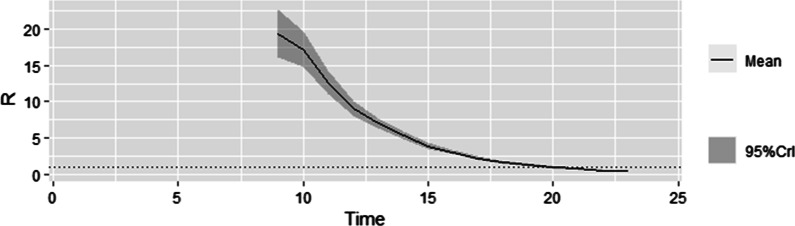
the distribution of likely values of time-dependent reproductive number during COVID-19 outbreak in Dalian during March 5 to April 2, 2022

The COVID-19 outbreak during March 14 to April 2, 2022: The earliest onset time of cases in this outbreak is March 5. The mean reproduction number estimate for the first seven days of the outbreak namely during March 5 to March 11 was 19.306 (95% CI:16.083–22.820). This was the maximum value of $R_{t}$. Subsequently the $R_{t}$_t_ trend began to decline (Fig. 2).

### Established the prediction model of $R_{t}$ and evaluated the prediction effect

We selected a section of data with a declining incidence trend as the basic data to fit the exponential trend equation. In this study, we used the value of $R_{t}$ with illness onset between July 19 to July 28, 2020, March 5 to March 17, 2022 to fit the prediction model, while the following value of $R_{t}$ were used to evaluate the quality of forecasting (Additional file 1). The equation R square were 0.982 and 0.980, respectively, all of them were greater than 0.85, the results showed that the exponential trend equation had a good fitting effect on the historical data.

In the COVID-19 outbreak during July 22 to August 5, 2020, the relationship was expressed in following equation:

7 $$y = 9.470e^{ - 0.16x} .$$

In the COVID-19 outbreak during March 14 to April 2 2022, the relationship was expressed in following equation:

8 $$y = 27.136e^{ - 0.268x} .$$

**Fig. 3:**
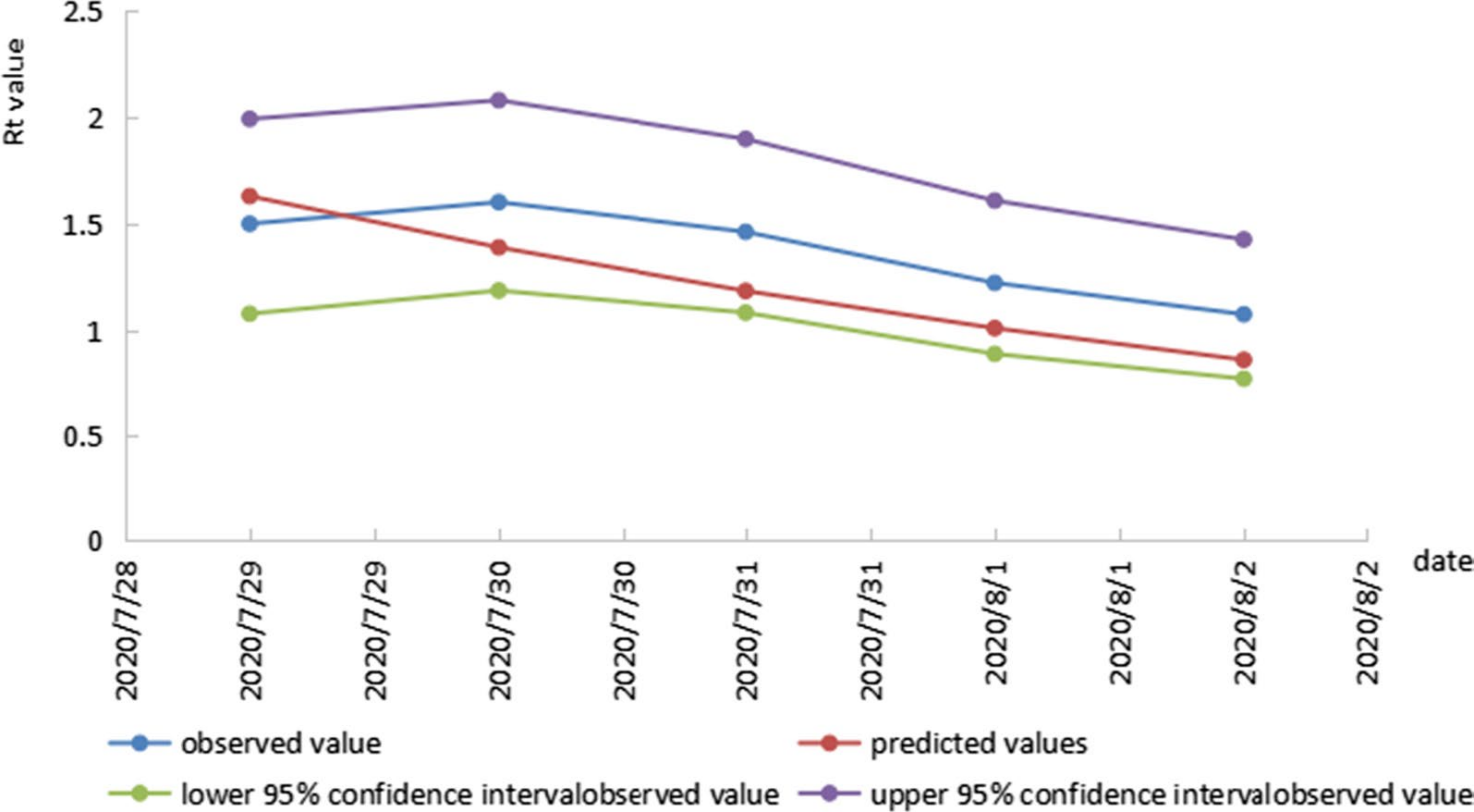
comparison of observed value of $R_{t}$ and predicted values obtained from exponential trend equation model during July 22 to August 5, 2020 COVID-19 outbreak in Dalian

**Fig. 4:**
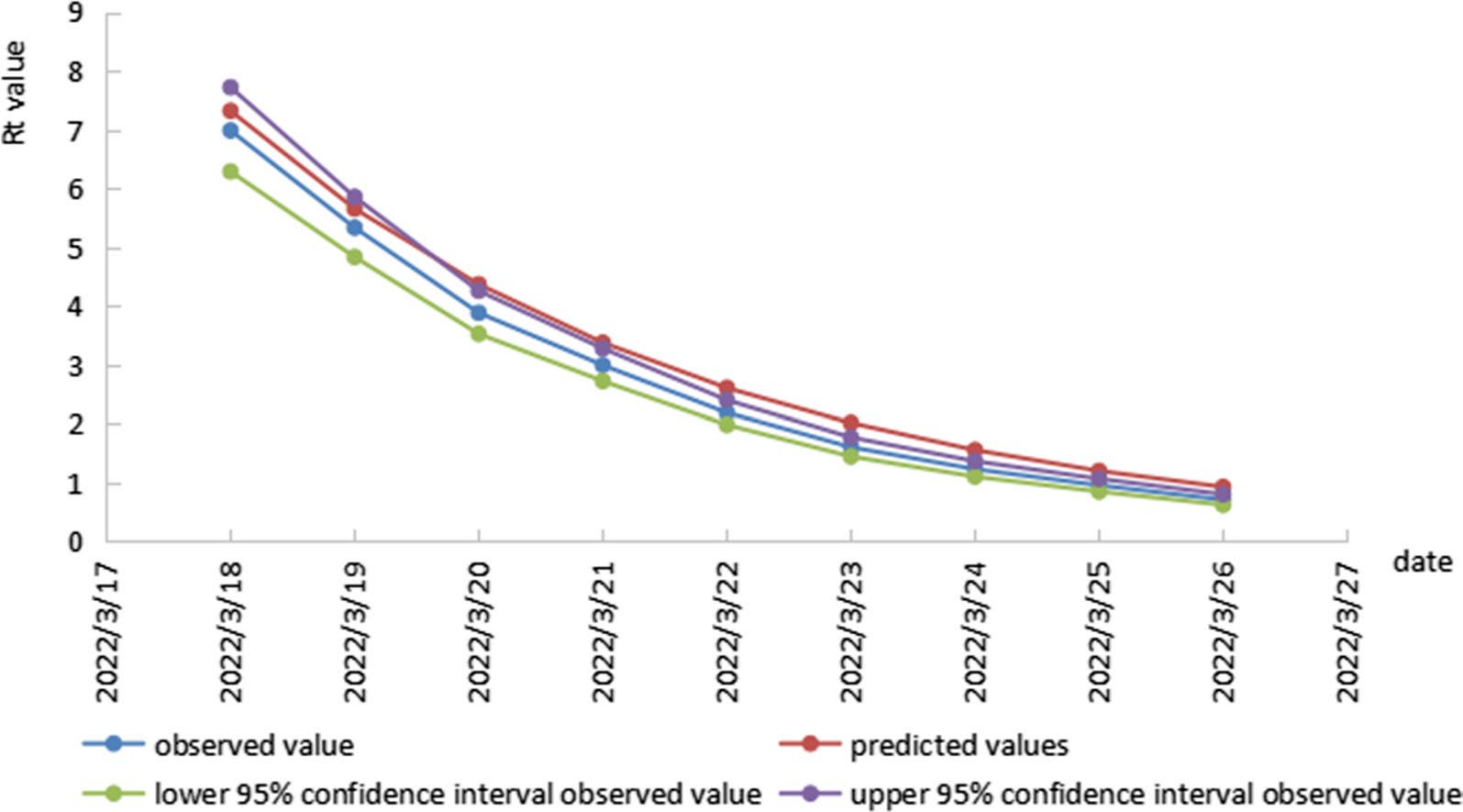
comparison of observed value of $R_{t}$ and predicted values obtained from exponential trend equation model during March 14 to April 2, 2022 COVID-19 outbreak in Dalian

We use above exponential trend equation to predicted Rt of the following data. Table 2, Figs. 3 and 4 shows the number of predicted $R_{t}$ from July 29 to August 2 and March 18 to March 26 obtained from the exponential trend equations. According to the result of prediction, under the current strength of prevention and control, the $R_{t}$ of COVID-19 would drop below 1 till August2, 2020, and March 26, 2022, respectively, which one day earlier or later than the actual date. The average relative error value for the model in the COVID-19 outbreak during July 22 to August 5, 2020 was − 12.21%. That in the COVID-19 outbreak during March 14 to April 2, 2022 was 18.14%.

**Table 2:** Comparison of observed value of $R_{t}$ and predicted values obtained from exponential trend equation models during two COVID-19 outbreaks in Dalian

Date	Predicted values	Observed value	95%CI	Relative error (%)
In the COVID-19 outbreak during July 22 to August 5, 2020				
2020/7/29	1.629	1.499	1.076–1.992	8.672
2020/7/30	1.388	1.601	1.185–2.080	− 13.304
2020/7/31	1.183	1.461	1.081–1.898	− 19.028
2020/8/1	1.008	1.221	0.887–1.608	− 17.445
2020/8/2	0.859	1.073	0.770–1.425	− 19.944
In the COVID-19 outbreak during March 14 to April 2, 2022				
2022/3/18	7.318	6.984	6.286–7.719	4.783
2022/3/19	5.654	5.332	4.833–5.854	6.037
2022/3/20	4.368	3.883	3.525–4.257	12.495
2022/3/21	3.375	2.993	2.723–3.277	12.758
2022/3/22	2.607	2.187	1.983–2.401	19.222
2022/3/23	2.014	1.605	1.447–1.771	25.511
2022/3/24	1.556	1.232	1.105–1.367	26.328
2022/3/25	1.202	0.957	0.851–1.068	25.646
2022/3/26	0.929	0.712	0.625–0.805	30.477

## Discussion

An outbreak of 2019 novel coronavirus diseases (COVID-19) in Wuhan, China has spread quickly nationwide, as of 10 am CETS August 16, 2020, a total of 212,94,845 confirmed cases and 761,779 deaths had been reported in 215 countries and regions on six continents [12]. From January 20, 2020 to August 5, 2020, 122 confirmed COVID-19 cases were reported in Dalian, including 12 imported cases from overseas. The above reported number of confirmed COVID -19 cases exceeded that in Jilin city [13] and less than that in Shanghai [14], Beijing [15], Guangzhou [16], Hangzhou [17], Tianjin [18] city in China.

In this study, to improve the goodness of fit, we selected a section of data with a declining incidence trend as the basic data to fit the exponential trend equation. According to the model's predictions, the turning point of above two COVID-19 outbreak in Dalian, Liaoning province, China will occur on August 2, 2020 and March 26, 2022.In other words, the epidemic situation has been effectively controlled in Dalian until August 2, 2020 and March 26, 2022 and the possibility of new cases being detected after August 2, 2020 and March 26, 2022 is low. If there are new cases, it indicates that there may be cases with long incubation period, new sources of infection or new transmission chains have been introduced.

The actual turning point of the this COVID-19 outbreak in Dalian, Liaoning province, China occurred on August 3,2020 and March 25,2022, one day later or earlier than the data obtained from the prediction model. The prediction effect of this study is better than the study result of Cui [19] et al. and Fan et al. [20] and worse than the study result of Lou et al. [21] and Liu [22]. Cui et al. used the Richards growth curve to predict the turning point based on the COVID-19 epidemic data in inland areas of China. This study concluded that the number of cases would decrease to double digits by mid-March 2020 (estimated after 7 March 2020) and to single digits by late March or early April 2020. The actual number of new cases dropped to double digits on March 6, and no new cases were reported after March 18. The predicted results were all later than the actual situation one day to 13 days. Fan et al. establish a SEIR dynamic model of the COVID-19 based on the complex network theory. This study concluded that the epidemic turning point of the Wuhan occurred between February 20 and 25, 2020.The actual maximum number of new cases in Wuhan on February 12.The predicted results were later than the actual situation 8 days to 13 days. Lou et al. used the Richards model to fit the cumulative number of COVID- 19 from January 10 to March 17, 2020 in Hubei Province and Wuhan City. This study estimated the outbreak turning point for COVID-19 in Wuhan to be 33.5 days with date of 02/12 (95%CI: 02/11–02/13), consistent with a wave peak in the number of new cases reported on the day of 02/12. Liu used the log linear regression model to find the influential factors of coronavirus transmission from the urban perspective and to predict the number of COVID-19 infection in Wuhan City. This study estimated number of COVID-19 infection cases in Wuhan city on March 2, 2020 was 56,944.866, which was very close to the officially reported number.

The exponential trend equation we developed gave an accurate estimate of $R_{t}$ values of COVID-19 for July29, July30 in 2020 and from March 18 to 21 in 2022, but the model failed to correctly estimate the $R_{t}$ of COVID-19 on July 31, August 1 and August 2, in 2020 and from March 22 to 26 in 2022 the relative error for them larger than 15% all. The reason for these error could be during July 6 to July 30 in 2020 and March 5 to March 21 in 2022 there were not only confirmed cases who have symptoms before diagnosis, but also included confirmed cases who have no any symptoms before diagnosis, whereas during July 31 to August 2 in 2020 and March 22 to 26 in 2022 there were only the second type of cases. For the second type of cases, the illness onset date equaled the date of diagnosis. If an asymptomatic infected person was diagnosed based on imaging features in the lungs, the date of diagnosis was related to the time of imaging tests, but the imaging tests were not performed daily, so for some patients, the date of diagnosis was not the actual date of illness onset. This is the one limitation of this study.

As a new respiratory infectious disease, on the one hand, COVID-19 is a new infectious disease, the population is generally susceptible to it, on the other hand, because it is a respiratory infectious disease, the transmission route is easy to realize, so COVID-19 is easy to lead to outbreak and epidemic. Once an outbreak occurs, through predicting turning points of COVID-19, we can know whether the current prevention and control measures are effective and whether it is necessary to adjust the prevention and control measures, which has important public health significance for reducing the total incidence during the outbreak. Therefore, predicting turning points of COVID-19 outbreak is not only important for Dalian, but also for other regions in the world.

When a local epidemic of COVID-19 occurs in a place in the world, the time-dependent reproduction number $R_{t}$ value can be calculated by combining the onset date data obtained from epidemiological investigation, and then the exponential regression equation can be fitted with the $R_{t}$ value as the basic data to predict when the $R_{t}$ value drops below 1. $R_{t}$ value less than 1 can indicates that the epidemic has been effectively controlled. If $R_{t}$ value which is in a downward trend, suddenly rises from one day, it indicates that a new sources of infection or new transmission chains have been introduced. By analyzing the trend of daily $R_{t}$ value, a sensitive early warning mechanism can be established to guide the timely adjustment of prevention and control strategies.

Not only in Dalian, but also in other regions of the world, as long as the incidence data of COVID-19 are available, the turning points of COVID-19 outbreak can be predicted by calculating the Rt value and fitting relevant models. It should be noted that we fitted the exponential regression model to the data of Dalian, and other models may be needed to fit the data of other regions.

The highlight the novelty and value of this study is $R_{t}$ value was used as the basic data to establish the prediction model, so as to understand when the epidemic was effectively controlled. This provides a new alternative method for predicting turning points of COVID-19 outbreak that is different from the aforementioned SEIR model, Richard model, and log linear regression model et al. Because only the onset date of the case is needed for Rt value calculation, the required data is easy to obtain, and exponential regression model is used to predict, so compared with other prediction models related to COVID-19, the prediction model is not only simple in structure, and easy to calculate, and the results are basically reasonable.

This study had another limitation. We didn’t predict when $R_{t}$ would reach its peak, which was also a very important issue and needs to be further studied (Additional file 1).

## Conclusions

In practice, the incidence number of cases calculated by the date of illness onset will vary from day to day, while the outbreak is not over. So the prediction results will change dynamically. However, the overall trend is consistent. The results of this study show that time-dependent reproduction number values fitted by exponential trend equation can used to predict the turning point of COVID-19 outbreak in Dalian, Liaoning province, China, and the results were consistent with the actual incidence on the whole, and the results can be used to establish a sensitive early warning mechanism to guide the timely adjustment of COVID-19 prevention and control strategies. This study not only may provide a new alternative method for predicting turning points of COVID-19 outbreak for the researchers in the field of infectious disease prediction, but also, may provide methods for policy makers how determine when an outbreak is under control and when need to adjust prevention and control measures.

## Supplementary Information

**Additional file 1. **Daily onset cases ofCOVID-19 in Dalian, Liaoning province, China, during July 9, 2020 to July 28,  2020 and March 14, 2022 to April 2, 2022.

## Data Availability

All data generated or analized during this study are included in this published article and its Additional files.

## Abbreviation

Rt: Time-dependent reproduction number
